# Optimizing charge transport in hybrid GaN-PEDOT:PSS/PMMADevice for advanced application

**DOI:** 10.1038/s41598-024-63197-3

**Published:** 2024-06-04

**Authors:** Makram A. Fakhri, Evan T. Salim, Marwah R. Ketab, Haneen D. Jabbar, Omar A. Ibrahim, Ahmad S. Azzahrani, Mohammed Jalal AbdulRazzaq, Raid A. Ismail, Ali Basem, Forat H. Alsultany, Subash C. B. Gopinath

**Affiliations:** 1 Laser and Optoelectronic Engineering Department, University of Technology-Iraq, Baghdad, Iraq; 2 Applied Science Department, University of Technology-Iraq, Baghdad, Iraq; 3 Iraqi Ministry of Health, Baghdad, Iraq; 4 Shatt Al-Arab University College, Basrah, Iraq; 5 Department of Physics, College of Science, University of Baghdad, Baghdad, Iraq; 6 Electrical Engineering Department, Northern Border University, Arar, Kingdom of Saudi Arabia; 7 Air Conditioning Engineering Department, Faculty of Engineering, Warith Al-Anbiyaa University, Karbala, 56001 Iraq; 8 Department of Medical Physics, Al-Mustaqbal University College, Babylon, Iraq; 9 Faculty of Chemical Engineering & Technology, Universiti Malaysia Perlis (UniMAP), 02600 Arau, Perlis Malaysia; 10 Institute of Nano Electronic Engineering, Universiti Malaysia Perlis (UniMAP), 01000 Kangar, Perlis Malaysia; 11 Center for Global Health Research, Saveetha Medical College & Hospital, Saveetha Institute of Medical and Technical Sciences (SIMATS), Thandalam, Chennai, Tamil Nadu 602 105 India

**Keywords:** Hybrid heterojunction, Light emitting diode, GaN, Pulsed laser deposition, Nanoscience and technology, Optics and photonics

## Abstract

Organic–inorganic hybrid light-emitting devices have garnered significant attention in the last few years due to their potential. These devices integrate the superior electron mobility of inorganic semiconductors with the remarkable optoelectronic characteristics of organic semiconductors. The inquiry focused on analyzing the optical and electrical properties of a light-emitting heterojunction that combines p-type GaN with organic materials (PEDOT, PSS, and PMMA). This heterojunction is an organic–inorganic hybrid. The procedure entailed utilizing a spin-coating technique to apply a layer of either poly(methyl methacrylate) (PMMA) or a mixture of PMMA and poly(3,4ethylenedioxythiophene)-poly(styrene sulfonate) (PEDOT: PSS) onto an indium tin oxide (ITO) substrate. Subsequently, different Nd:YAG laser pulses (200, 250, and 300 pulses) were used to administer a GaN inorganic layer onto the prepared organic layer using a pulsed laser deposition approach. Subsequently, the thermal evaporation technique was employed to deposit an aluminum electrode on the top of the organic and inorganic layers, while laser pulses were fine-tuned for optimal performance. The Hall effect investigation verifies the p-type conductivity of the GaN material. The electroluminescence studies confirmed the production of blue light by the GaN-based devices throughout a range of voltage situations, spanning from 45 to 72 V.

## Introduction

Gallium Nitride (GaN) is one of the most promising inorganic III-semiconductors for optoelectronic devices because it has a wide bandgap, high electron mobility, and great thermal stability. It has been employed in applications involving high-temperature, high-power electronic devices^1–5^. However, a problem arises due to the absorption of phosphor by GaN organic material, resulting in a decrease in down-conversion efficiency and constraints in color rendering^6–13^. Another obstacle involves addressing the intricate and costly fabrication process^11,14–16^.

On a different note, organic light-emitting diodes demonstrate the capability to cover the visible spectrum, including ultraviolet and near-infrared wavelengths, offering remarkable brightness efficiency and faster response times^17–20^. However, the downside lies in the electrical and stability issues associated with organic semiconductors^21–24^. Consequently, the integration of organic–inorganic hybrid light-emitting devices emerges as a solution, combining the outstanding electrical features of inorganic semiconductors with the exceptional optoelectronic properties of organic materials^25–31^.

A lot of attention has been paid to GaN-based organic–inorganic hybrid light-emitting hetero-junctions as a new type of material for high-performance light-emitting diodes (LEDs) and other optoelectronic devices^32–36^. These hetero-junctions combine the advantages of both organic and inorganic materials, resulting in improved device performance and the potential to replace traditional lighting^11,37,38^. Numerous research groups have reported on the development of GaN-based organic–inorganic heterojunctions for LEDs. Yang et al.^39^ looked into the features and performance of an n-type GaN-based mixed organic and inorganic light-emitting heterojunction. The researchers manufactured the hybrid heterojunction by employing a layer-by-layer deposition technique and analyzed its structural, optical, and electrical characteristics^39^. The findings showed that the hybrid heterojunction could switch on and off, produce electroluminescence well (with a peak emission wavelength of about 520 nm), have a low turn-on voltage, and be very bright. These results suggest its potential as a promising light-emitting device^40–44^.

In this work, an organic–inorganic hybrid light-emitting heterojunction was fabricated by combining p-type GaN with organic materials (PEDOT: PSS, and PMMA) and investigating its optical and electrical properties. The spin-coating method was employed to put an organic layer of PMMA or a mix of PMMA and PEDOT:PSS onto a base made of indium tin oxide (ITO). Next, apply an inorganic layer of GaN to the prepared organic layer using the pulsed laser deposition technique with different Nd:YAG laser pulses, specifically 200, 250, and 300 pulses. In this work, the effect of laser pulse number on the performance of the prepared optoelectronics device was investigated, which, to the best of our knowledge, has not been extensively studied yet.

## Steps of work

### Preparation and characterization of ITO/PMMA and ITO:PEDOT: PSS/PMMA substrates

The researchers used an indium tin oxide (ITO) glass substrate to provide transparency and employed PEDOT:PSS to enhance the matching between ITO glass and PMMA. Additionally, 250 μl of poly-methyl methacrylate (PMMA) from “United States, Sigma Aldrich Co.” is used. Before the fabrication of the hybrid film, the ITO glass substrate was cleaned in an ultrasonic bath containing a solution of water and liquid soap for 30 min.

The PMMA solution was prepared by dissolving 3 g of it in 100 ml of 99% concentrated chloroform. We filtered the PEDOT:PSS compound through a 0.45 μm PES filter into an amber vial and stored it in a container approved for long-term exposure to light.

A spin coating system was used to prepare the ITO: PMMA film on the ITO glass substrate using a 250 μl PMMA solution at 2500 rpm for 30 s.

**Figure 1:**
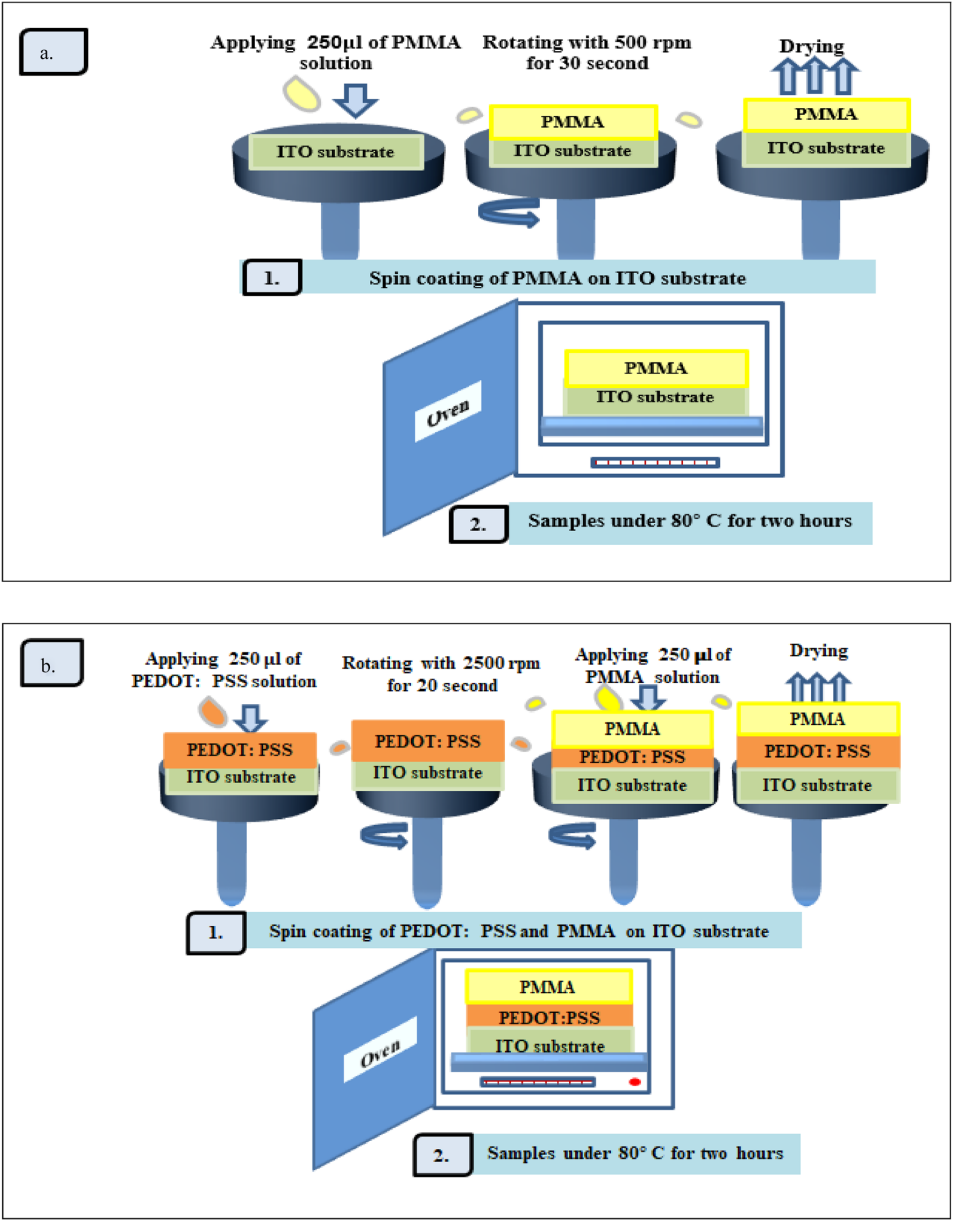
(**a**,**b**) Preparation of ITO:PMMA film, ITO:PEDOT: PSS:PMMA respectively.

The ITO:PMMA substrate was prepared by spin-coating the ITO glass substrate with a 250 μl PMMA solution at 2500 rpm for 30 s. Then, we annealed it at 80 °C for 2 h to remove the solvent, as shown in Fig. 1a. Additionally, the ITO:PEDOT:PSS:PMMA composite was prepared in the same manner, as shown in Fig. 1b. We prepared PSS: PMMA in the same manner as depicted in Fig. 1b.

### Deposition of inorganic GaN material on organic substrates

**Figure 2:**
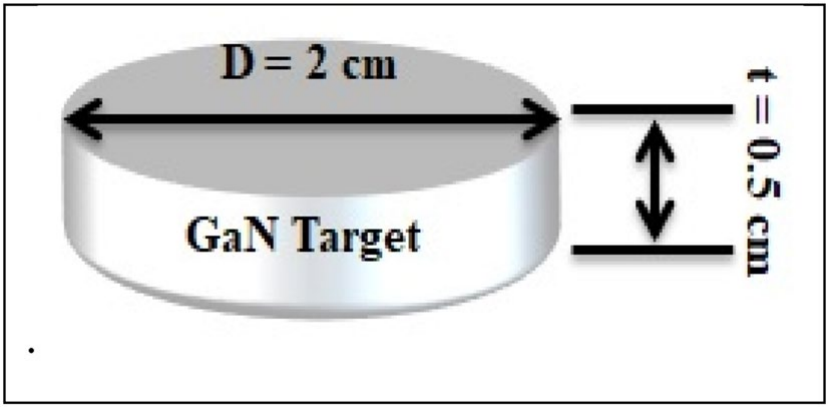
GaN target dimensions.

High-purity gallium nitride powder (99.9%), obtained from Luoyang Advanced Material Corporation, China, was pressed using a hydraulic press at 15 kg/cm^2^ to prepare a 2 cm diameter and 0.5 cm thickness GaN target, as shown in Fig. 2.

### Deposition of inorganic GaN material on organic substrates

**Figure 3:**
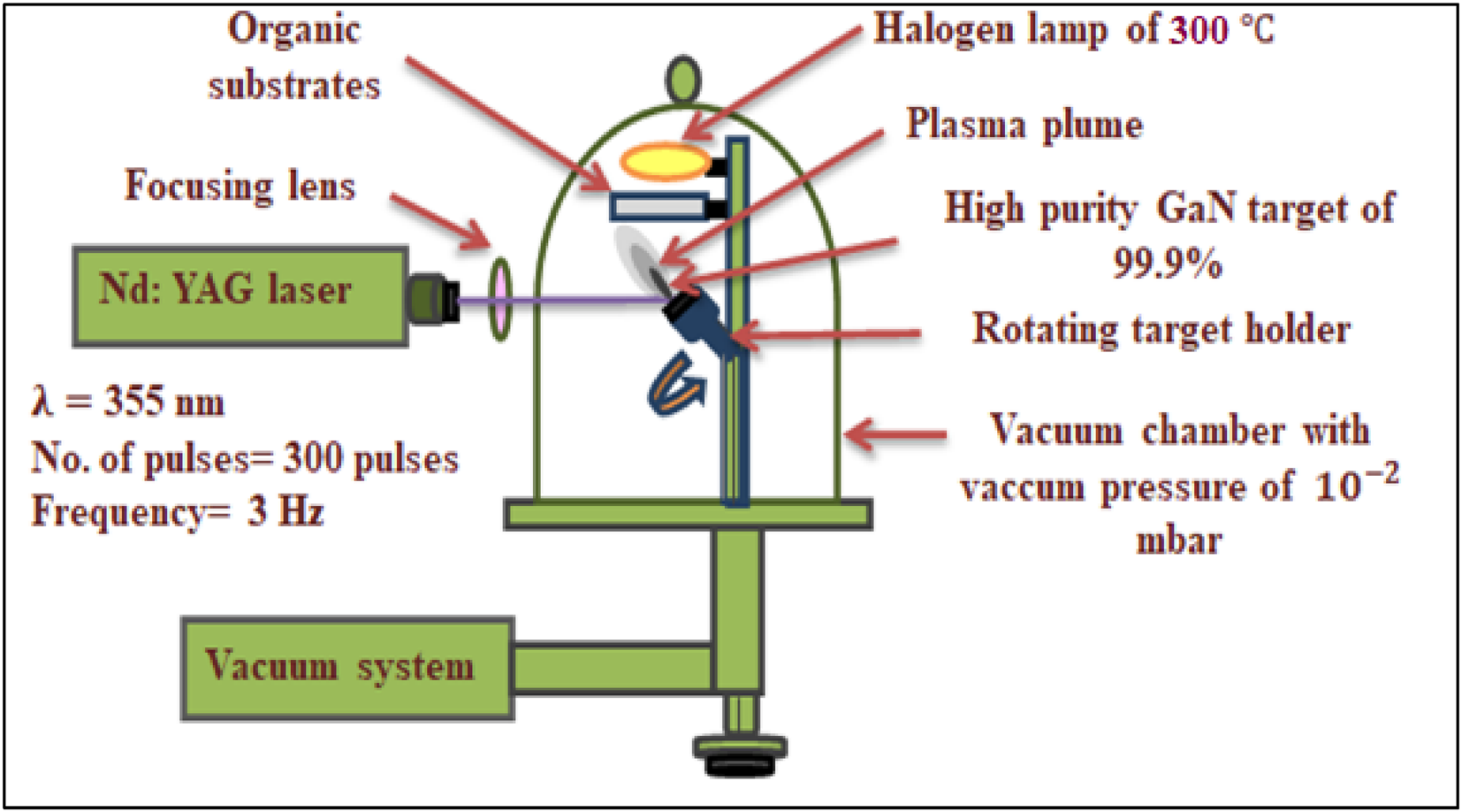
Deposition steps of inorganic GaN on ITO: PMMA substrates with different laser pulses and ITO: PEDOT:PSS: PMMA organic substrate with optimum laser pulses.

A 7.95 mJ/cm^2^ fluence and 355nm wavelength of Q-switched Nd:YAG laser from RY 280, China was used to deposit GaN nanostructure thin film on the chemically prepared substrate with a different number of laser pulses (200, 250, and 300 pulses) to prepare ITO:PMMA:GaN and ITO:PEDOT:PSS:PMMA hybrid junctions, as illustrated in Table 1. The pulsed laser deposition system (PLD) can be shown in Fig. 3.

**Table 1:** Laser deposition parameters of GaN inorganic material over ITO: PMMA substrates and ITO:PEDOT:PSS: PMMA organic substrate.

Deposition parameters	The values
Nd: YAG laser wavelengths	355 nm
Nd:YAG laser energy	900 mJ 7.95 mJ/cm^2^
Pulse duration	7 ns
Frequency	3 Hz
Repetition rate	200, 250, and 300 pulses
Spot size	1 mm
The focal length of the lens	12 cm
Power supply	220 V
Substrate	Organic
Substrate temperature	300 $^\circ{\rm C}$
Cooling conditions	Air and water

The fabrication of GaN-based organic–inorganic LEDs involves a comprehensive series of tests and experiments to optimize results for various laser pulses. For the investigation of material absorption, a UV–visible spectrophotometer (SP-8001) from China with a wavelength range of 190–1100 nm, equipped with a deuterium and halogen lamp that automatically switches at 320–360 nm, was employed. Spectroscopic properties were examined using photoluminescence (PL) equipment from the American company PerkinElmer. Additionally, electroluminescence (EL) measurements were conducted using a device (RF-5301PC) from the Japanese Shimadzu company. Furthermore, I-V characteristics were analyzed using a power supply (Dazheng from China, up to 80 V, 5 A, PS-305D) and digital multimeters (UNI-T-UT33 and TEKR CDM 250).

### Ohmic contacts fabrication

**Figure 4:**
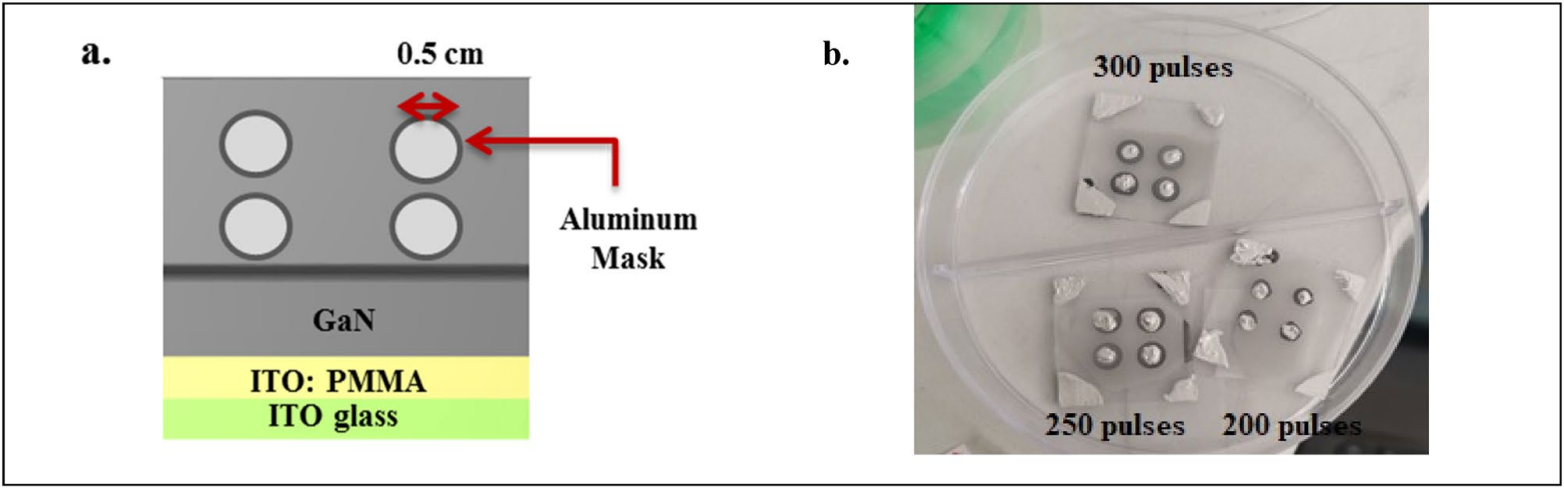
Fabricated ITO:PMMA: GaN hybrid junctions with aluminum mask. (**a**) Schematic diagram, (**b**) experimental image.

**Figure 5:**
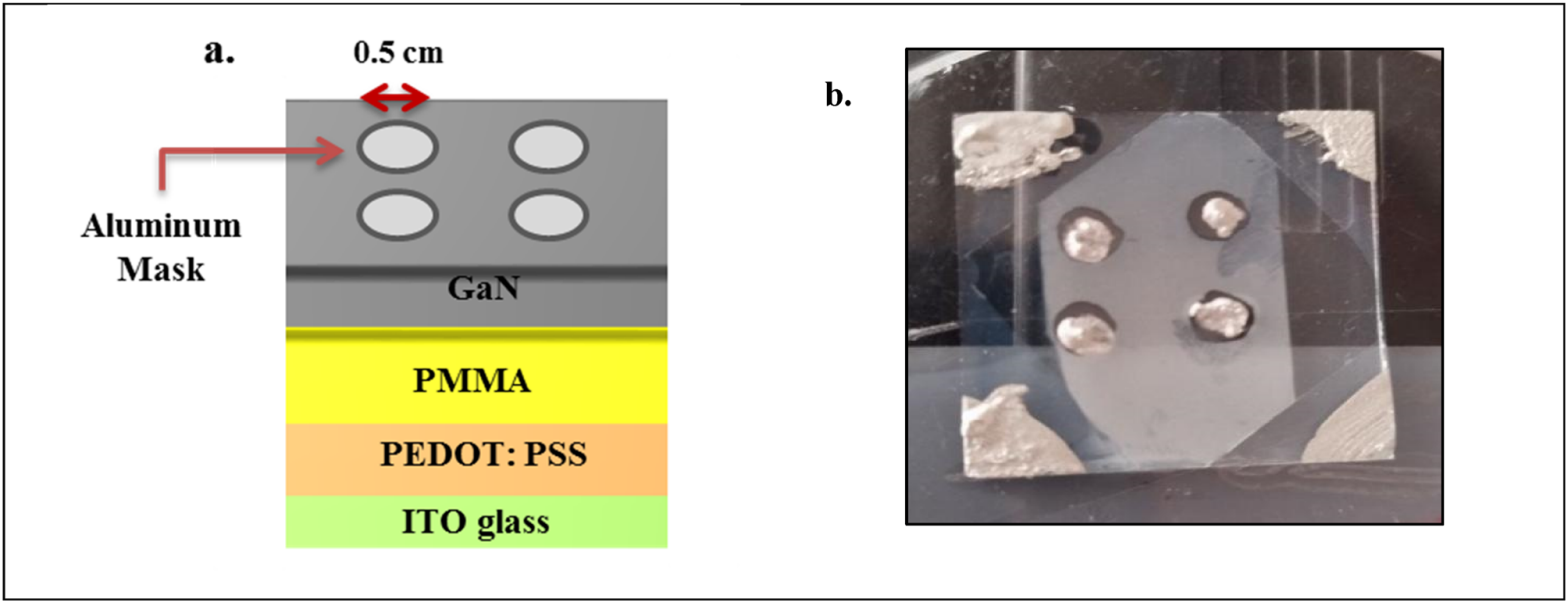
Fabricated ITO: PEDOT: PSS: PMMA: GaN hybrid junction under optimum laser pulses with an aluminum mask. (**a**) Schematic diagram; (**b**) experimental image.

Figures 4 and 5 illustrate the outcomes of depositing an Al electrode on the fabricated GaN hybrid junctions using a thermal evaporation system having 5 × 10^–5^ mbar pressure in the deposition chamber. A robust current gradually heated a resistive spiral of pure aluminum wire and a silver rod to a temperature at which aluminum vaporized, transforming into a gaseous state. The pre-treated GaN hybrid junctions collected atoms (molecules) from the evaporation source. We positioned an aluminum sheet mask, resembling a fingerprint mask, over the fabricated GaN hybrid junctions to create masks that define the geometry of the electrodes.

## Results and discussion

### GaN thin film investigation 3.1.1 atomic force microscopy (AFM) GaN films

**Figure 6:**
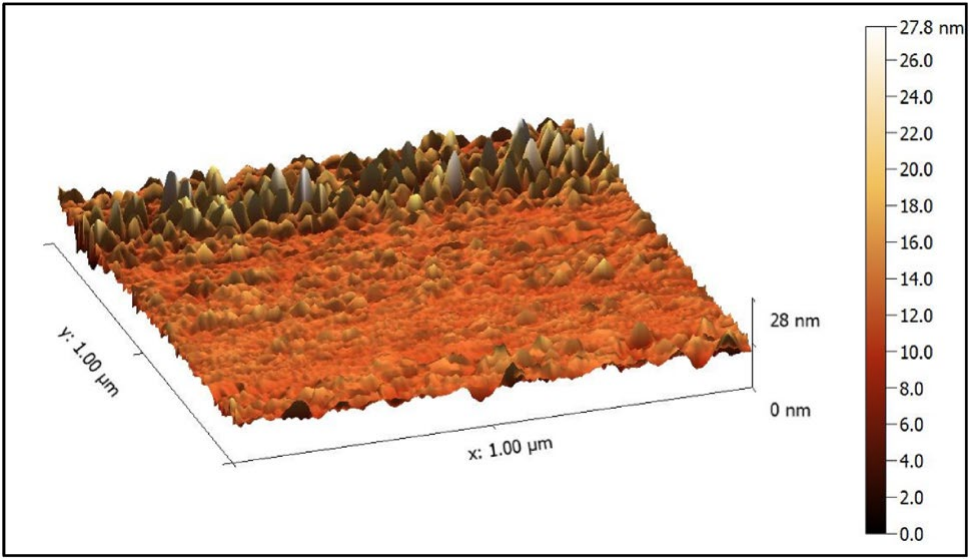
Atomic force microscopic results (AFM) of prepared GaN films.

Figure 6 shows three-dimensional AFM images and the size distribution of the grains in GaN nanofilms fabricated at 300 °C substrate temperatures. The smooth, uniform, and fine surface roughness could be recognized. The root mean square is 39.04 (nm), the average surface roughness is 20.15 (nm), and the thickness of the film is found to be about 2.426 μm^8,11,45,46^.

#### Field emission scanning electron microscopy (FESEM) of GaN film

**Figure 7:**
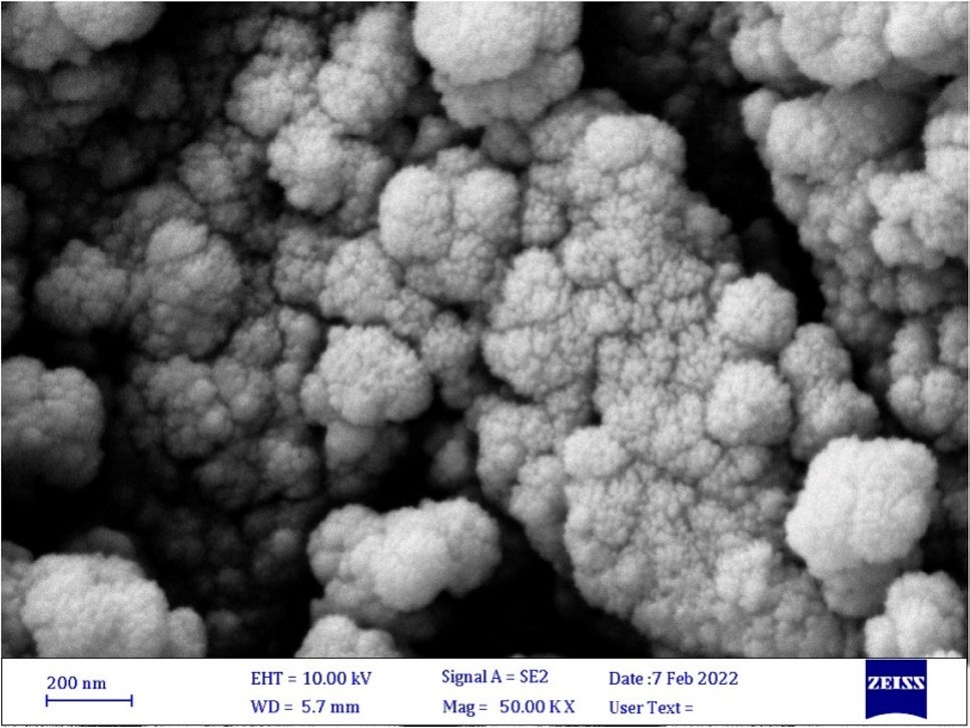
FE-SEM of used GaN thin films.

Figure 7 shows a field emission scanning electron micrograph (FESEM) of the employed GaN film taken before depositing ITO: PMMA and ITO:PEDOT: PSS: PMMA substrates. The picture reveals the surface morphology of prepared films on a nanoscale. The resulting GaN nanoparticles completely covered the substrate, forming spherical particles of uniform and homogeneous size, resembling the shape of cauliflower-like morphology^8,11,47,48^.

### Optical properties

#### Optical absorption

**Figure 8:**
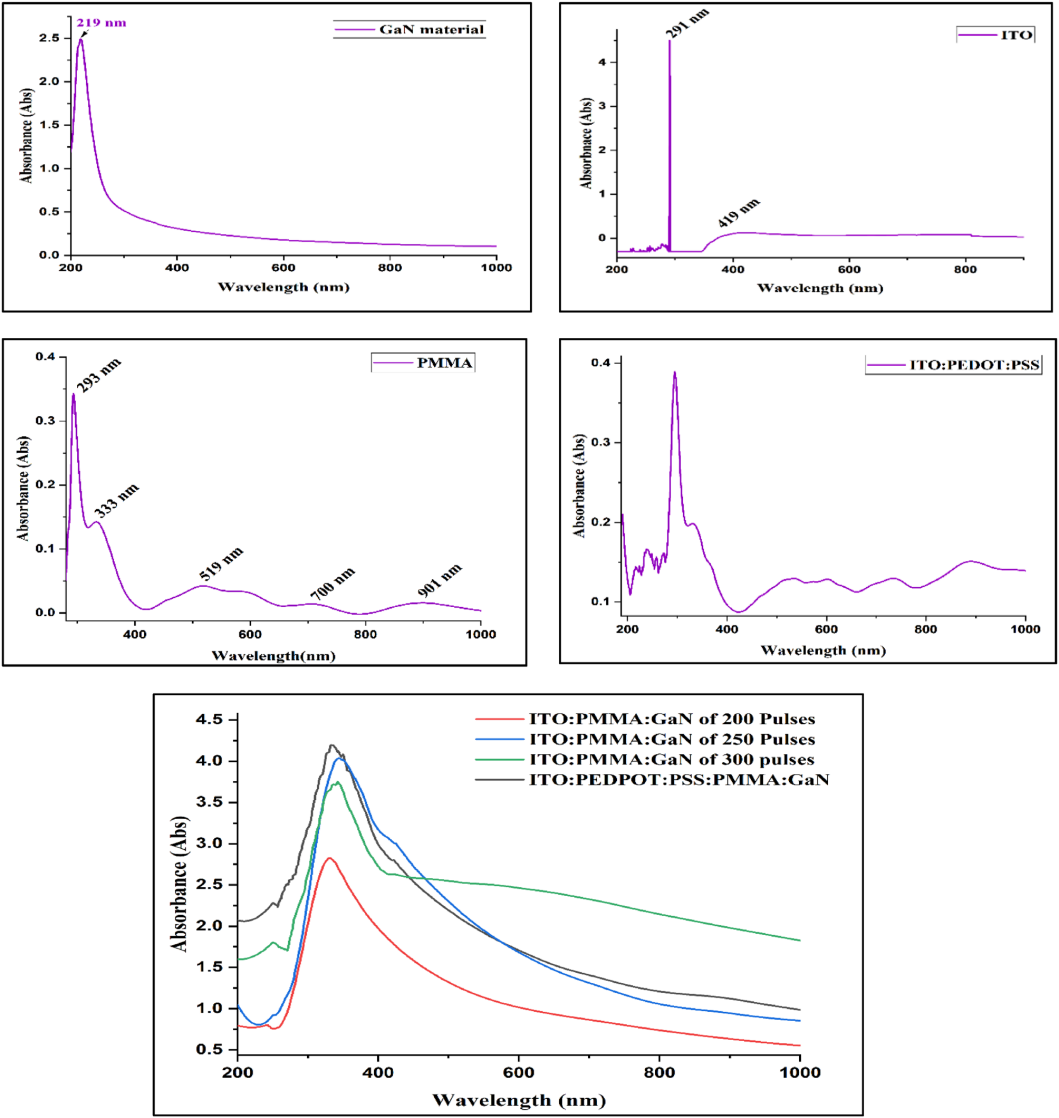
Optical absorbance plots of organic–inorganic materials and fabricated hybrid junction LEDs.

Figure 8 illustrates the absorption as a function of wavelength. In fact, the GaN thin film exhibits significant absorbance in the UV region, particularly at 219 nm, which decreases exponentially with increasing wavelength^49,50^**.**

#### Photoluminescence (PL)

**Figure 9:**
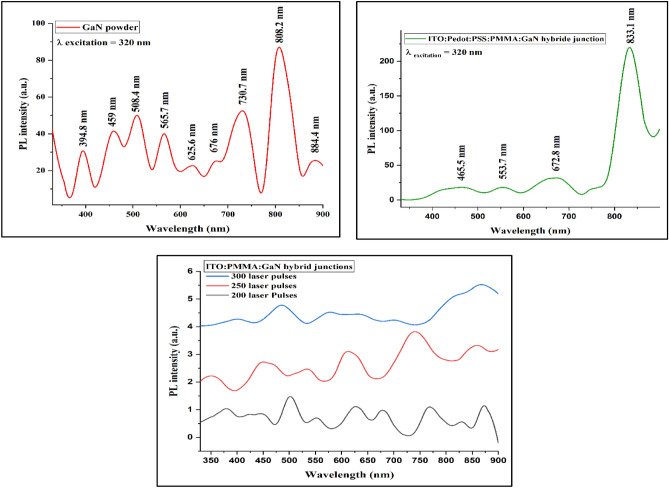
The PL spectra of fabricated ITO: PMMA: GaN hybrid junctions deposited at different numbers of laser pulses, and ITO: PEDOT: PSS:PMMA: GaN hybrid junction under optimum laser pulses.

Figure 9 shows the PL spectrum of GaN nanomaterial excited by a 320 nm wavelength. It is observed that the GaN material exhibits several PL peaks in the UV, visible, and IR regions. Figure 9 shows the observation of the PL spectrum in the blue, green, and red bands for the ITO:PEDOT:PSS:PMMA:GaN hybrid junction. Additionally, the PL spectrum of ITO:PMMA:GaN hybrid junctions at different laser pulses was observed in the UV, blue, green, orange, red, and IR, as shown in Fig. 9. We observed a blue shift in the PL spectrum of the ITO:PMMA:GaN hybrid junctions as the laser pulses increased. Increasing the number of laser pulses leads to a slight increase in FWHM and a decrease in the intensity of the PL peaks. This can be attributed to increasing the particle size, which comes from the agglomeration of the nanograins and the formation of some droplets^51–53^.

### Electrical characteristic

#### Hall effect

**Figure 10:**
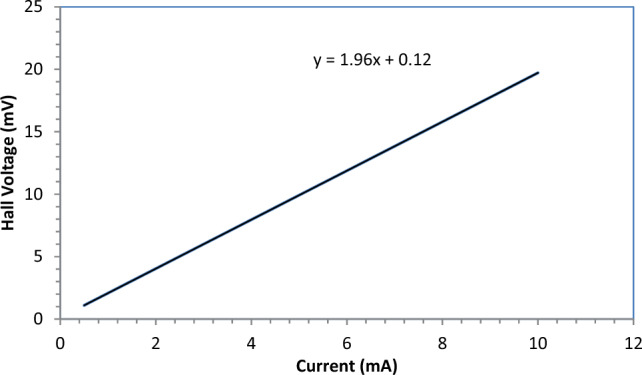
Hall effect characteristics of fabricated GaN for 300 laser pulses.

Two ohmic contacts were attached to the GaN layer and the substrate. Here, the current–voltage characteristic shown in Fig. 10 shows an almost ohmic behavior at the GaN interface, suggesting a strong dynamical exchange interaction between the magnetization in the GaN layer and carrier spins in the substrate layer.

We can easily identify whether a semiconductor is p-type or n-type by using the Hall Effect. If the voltage produced is positive, then the material is said to be p-type, and if the voltage produced is negative, then the material is said to be n-type^54,55^.

Hall measurements confirmed that GaN has a negative Hall coefficient, indicating that this material is p-type, as shown in Table 2.

**Table 2:** The Hall effect measurements of the GaN materials.

n (cm^−3^)	RH (cm^−3^ C^−1^)	µ (cm^2^/V s)	Type
7.41E^+13^	8.42E^+04^	8.68E^+03^	P

#### Current–voltage characteristics

**Figure 11:**
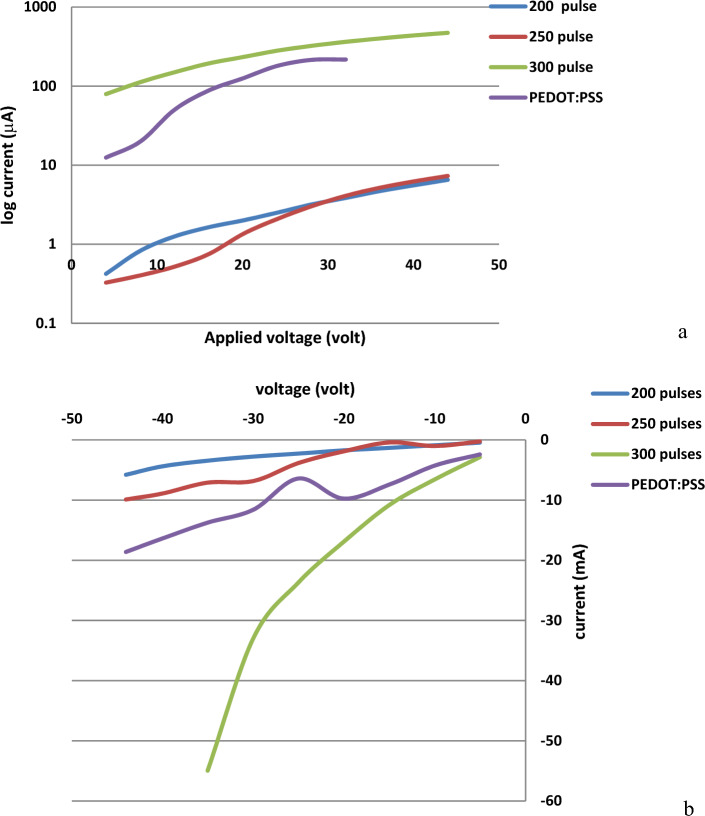
Current–voltage characteristics of fabricated GaN based on organic–inorganic hybrid junctions. (**a**) Forward, (**b**) reverse with different laser pulses.

The room-temperature I-V characteristics of the ITO:PMMA:GaN hybrid junction devices fabricated using the PLD technique at various numbers of laser pulses are shown in Fig. 11a,b. The forwarded current from the ITO: PMMA:GaN hybrid junctions was expected to exponentially increase with laser pulse numbers up to 300 pulses. The maximum achieved current was found to reach about 7 µA at 200 laser pulses, 8 μA for the junction prepared at 250 pulses, and 750 μA for the junction prepared at 300 laser pulses. Increasing the number of laser pulses leads to increased current due to decreasing the electrical resistivity of the GaN film since the film thickness increases directly with the laser pulses^56–58^.The 300 pulse has been chosen for the ITO:PEDOT:PSS:PMMA:GaN hybrid junction fabrication, as shown in Fig. 11a. Additionally, the obtained flow current from the ITO:PEDOT:PSS:PMMA:GaN hybrid junction was measured to be about 230 μA. Comparing the obtained current using 300 laser pulses and the current obtained from the hybrid junction, a clear reduction from ~ 700 to ~ 150 µA for 40 V, could be recognized, the reduction in current value may related to the film uniformity in the PEDOT:PSS layer which could create a regions with poor electrical conductivity, leading to increased resistance and lowering the current. In addition to above film thickness also may effected the current vale where an excessively thick PEDOT:PSS layer might increase the overall resistance of the device, thereby reducing the current, as it’s well known that using PLD technique may produce non uniform film thickness due to sublimation effect. One more reason could the Couse of current reduction which is the interface between PEDOT:PSS and PMMA or between PEDOT:PSS and ITO might have lattice mismatch that introduce trap states, increasing recombination and reducing current.

To estimate the current rectification ratio, the current of the prepared devices was measured under the reverse bias condition. The maximum rectification ratio was achieved for the device prepared using 300 laser pulses. In this case, the minimum reverse current (negative current) was found to be 10 mA at a 20 V negative voltage, as shown in Fig. 11b.

#### Electroluminescence (EL)

**Figure 12:**
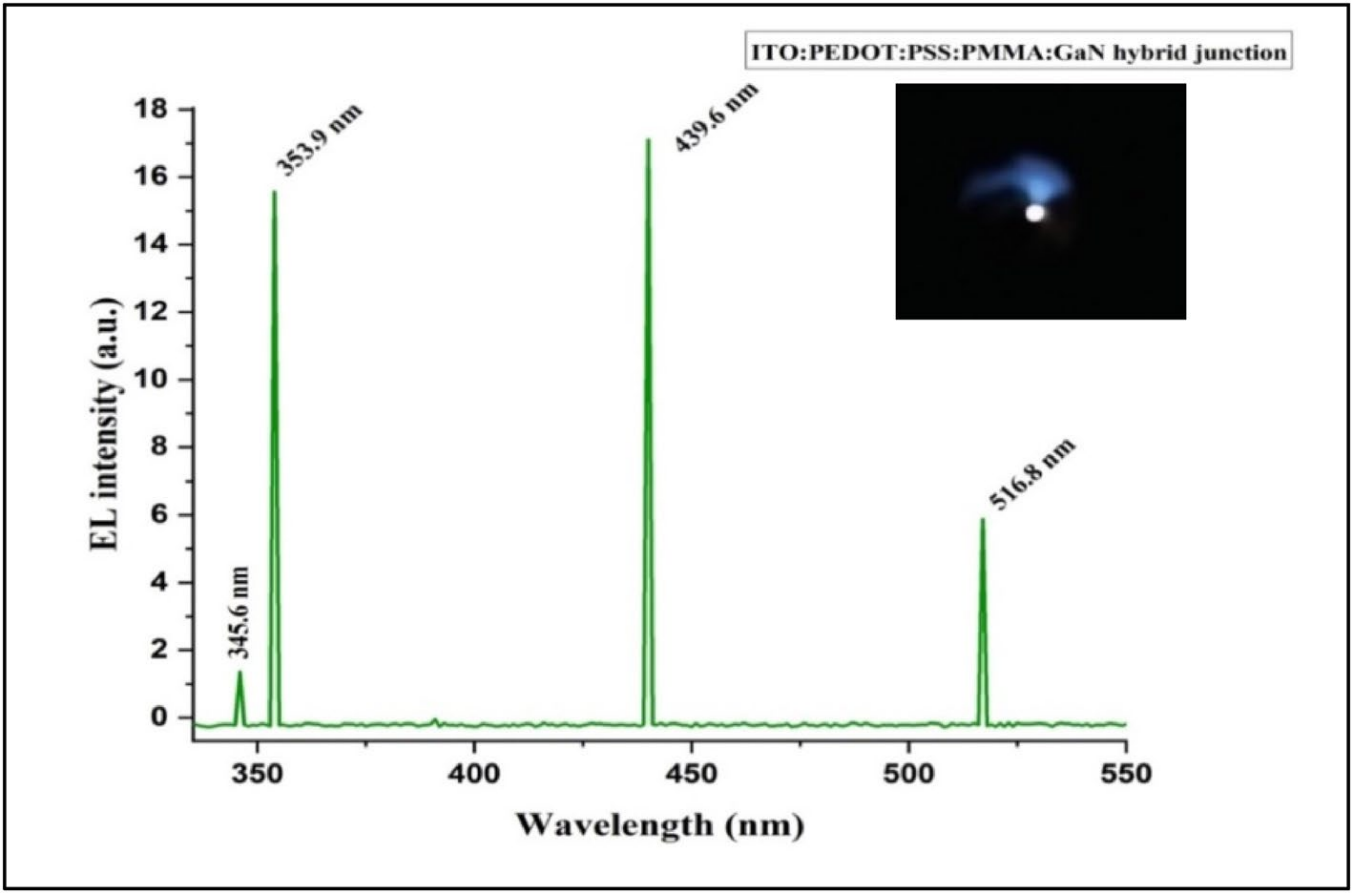
EL spectrum of a fabricated ITO: PEDOT:PSS:PMMA:GaN hybrid junction via the PLD method under 300 laser pulses.

**Figure 13:**
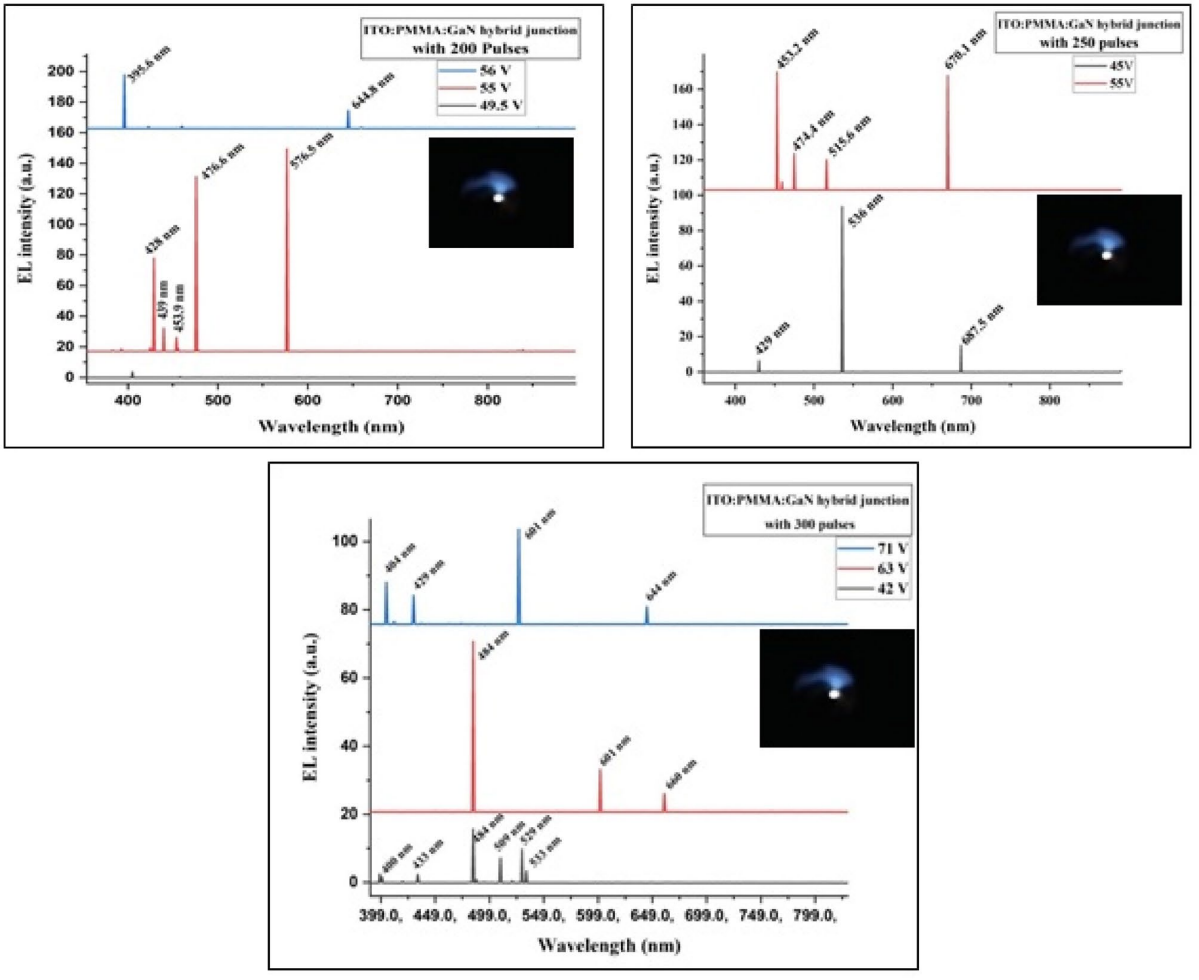
EL spectrum of fabricated hybrid junctions via PLD method with different laser pulses.

At room temperature, the EL measurements were performed, applying a forward bias voltage of 65 V to the ITO:PEDOT:PSS:PMMA:GaN hybrid junction device. We obtained the EL results using a photomultiplier detector, as shown in Figs. 12 and 13. ITO absorbed peaks at 394.8 nm, 730.7 nm, 808.2 nm, and 884.4 nm, which were not present in the EL spectrum of all fabricated hybrid junctions. This absorption can be attributed to the interference between the valence band and conduction band of GaN material and the homo and lumo bands of PMMA^59–64^.

The PL intensity peaks of the GaN material at 508.4 nm, 730 nm, and 808.2 nm were not evident in the EL spectrum of the ITO. PEDOT:PSS:PMMA:GaN hybrid junction due to high absorption by the PMMA layer at 519 nm and 700 nm. It's noteworthy that the maximum peak at 808.2 nm does not appear in the EL spectrum, despite having high intensity, owing to the PMMA layer's significant absorption in the IR region, as illustrated in Fig. 8.

## Conclusion

Using a p-type inorganic GaN material that we created in this study to make inorganic–organic hybrid junctions using the pulsed laser deposition method with 200, 250, and 300 pulses of laser light. All GaN-based organic–inorganic hybrid junctions demonstrate blue light emission, attributed to efficient hole injection. Additionally, the Hall effect investigation reveals that the GaN type is p-type. Moreover, the GaN fabricated on an ITO: PMMA substrate using the PLD method with 300 laser pulses exhibited the best photoluminescence (PL), electroluminescence (EL), and I-V characteristics among the fabricated hybrid junction LEDs.

## Data Availability

Correspondence and requests for materials should be addressed to Makram A. Fakhri, Haneen D. Jabbar, and Evan T. Salim.
